# Supplementary material for: Point Mutations of Nicotinic Receptor α1 Subunit Reveal New Molecular Features of G153S Slow-Channel Myasthenia
Source: Molecules. 2021 Feb 26;26(5):1278. doi: 10.3390/molecules26051278 (PMC7956382; doi:10.3390/molecules26051278)
Supplement: Supplementary file 1 [file molecules-26-01278-s001.pdf]

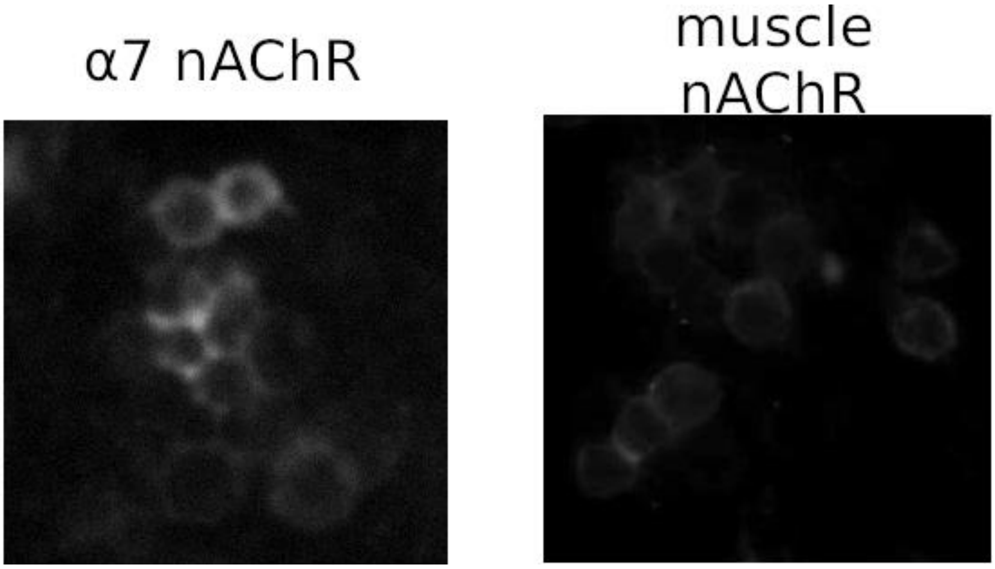

Figure S1. Comparison of α7 nAChR expression co-expressed with NACHO and Ric-3 chaperone proteins. Cells are stained with Alexa Fluor 555 α-bungarotoxin (100 nM).

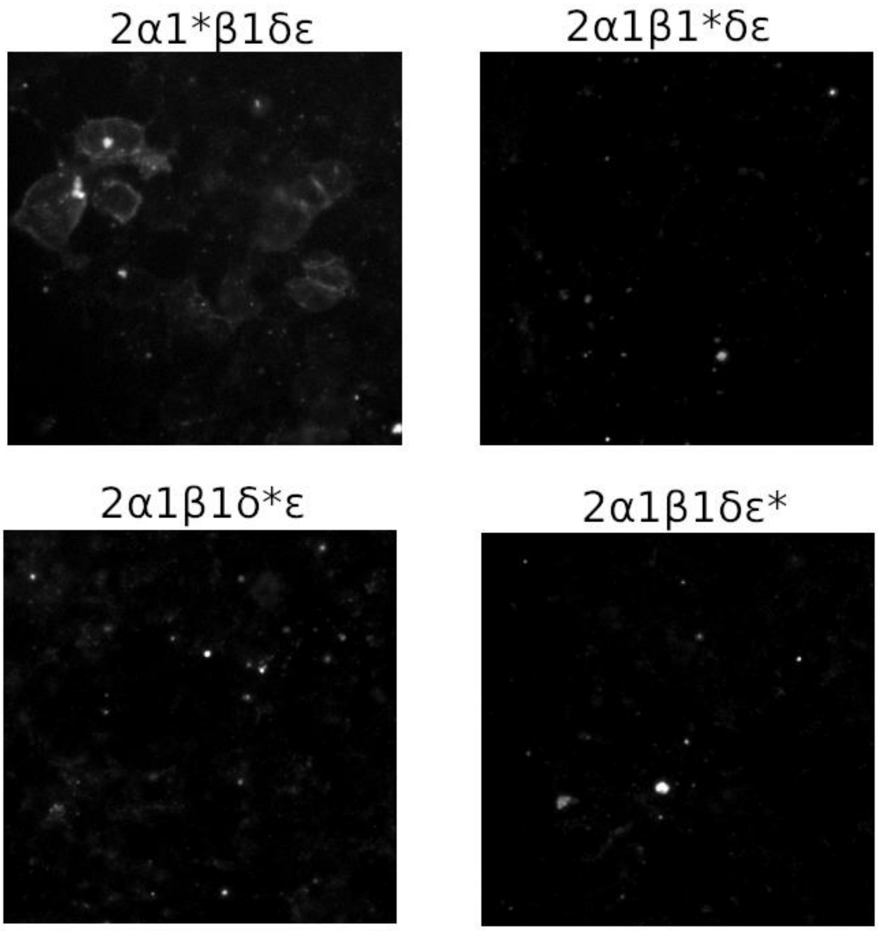

Figure S2. Expression of diverse muscle nAChR subunit combinations, demontrating that all four muscle nAChR subunits (α1, β1, δ and ε) is needed to produce detectable amounts of surface expressed receptors. Asterisc denotes the mutated non-expressing subunit gene used in the transfection protocol.
